# In Situ Electrochemical Mn(III)/Mn(IV) Generation of Mn(II)O Electrocatalysts for High-Performance Oxygen Reduction

**DOI:** 10.1007/s40820-020-00500-7

**Published:** 2020-08-11

**Authors:** Han Tian, Liming Zeng, Yifan Huang, Zhonghua Ma, Ge Meng, Lingxin Peng, Chang Chen, Xiangzhi Cui, Jianlin Shi

**Affiliations:** 1 State Key Lab of High Performance Ceramics and Superfine Microstructure, Shanghai Institute of Ceramics, Chinese Academy of Sciences, Shanghai, 200050 People’s Republic of China; 2 Center of Materials Science and Optoelectronics Engineering, University of Chinese Academy of Sciences, Beijing, 100049 People’s Republic of China; 3 School of Chemistry and Materials Science, Hangzhou Institute for Advanced Study, University of Chinese Academy of Sciences, Hangzhou, 310021 People’s Republic of China; 4 College of Chemistry and Molecular Sciences, Hubei Key Lab of Electrochemical Power Sources, Wuhan University, Wuhan, 430072 People’s Republic of China; 5 Wuhan University of Science and Technology, Wuhan, 430081 People’s Republic of China; 6 College of Material Science and Engineering, Donghua University, Shanghai, 201620 People’s Republic of China

**Keywords:** Zinc–air battery, In situ generation, High-valence manganese species, Synergetic catalytic effect

## Abstract

**Highlights:**

- MnO rich in oxygen vacancies has been synthesized.
- The synthesized MnO demonstrates excellent oxygen reduction reaction performance and high output power in Zn–air battery.
- The high catalytic activity is attributed to the synergetic catalytic effect between oxygen vacancies and in situ generated Mn^3+^/Mn^4+^.

**Abstract:**

Among various earth-abundant and noble metal-free catalysts for oxygen reduction reaction (ORR), manganese-based oxides are promising candidates owing to the rich variety of manganese valence. Herein, an extremely facile method for the synthesis of cubic and orthorhombic phase coexisting Mn(II)O electrocatalyst as an efficient ORR catalyst was explored. The obtained MnO electrocatalyst with oxygen vacancies shows a significantly elevated ORR catalytic activity with a half-wave potential (*E*_1/2_) of as high as 0.895 V, in comparison with that of commercial Pt/C (*E*_1/2_ = 0.877 V). More impressively, the MnO electrocatalyst exhibits a marked activity enhancement after test under a constant applied potential for 1000 s thanks to the in situ generation and stable presence of high-valence manganese species (Mn^3+^ and Mn^4+^) during the electrochemical process, initiating a synergetic catalytic effect with oxygen vacancies, which is proved to largely accelerate the adsorption and reduction of O_2_ molecules favoring the ORR activity elevation. Such an excellent ORR catalytic performance of this MnO electrocatalyst is applied in Zn–air battery, which shows an extra-high peak power density of 63.2 mW cm^−2^ in comparison with that (47.4 mW cm^−2^) of commercial Pt/C under identical test conditions.
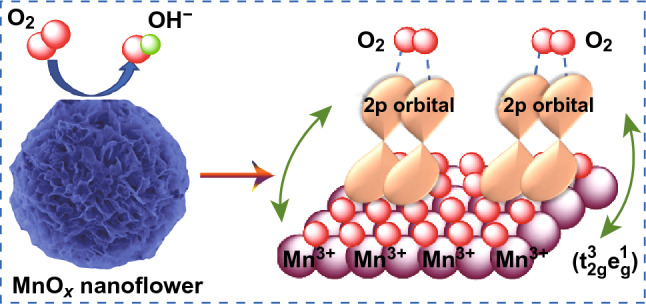

**Electronic supplementary material:**

The online version of this article (10.1007/s40820-020-00500-7) contains supplementary material, which is available to authorized users.

## Introduction

Advanced energy storage and conversion systems, such as fuel cells and metal–air batteries, are attracting more and more attentions worldwide due to the ever-increasing fossil energy consumption and accompanying severe environmental problems [1, 2]. Great progresses have been made in metal–air batteries (especially Zn–air battery) thanks to the simplicity in cell structure and operation [3, 4], and more importantly, the opportunities of employing a large variety of noble metal-free catalysts. However, the thermodynamically sluggish oxygen reduction reaction (ORR) kinetics and severe energy efficiency losses during the cell operation remain the main obstacles for the commercialization of this kind of metal–air batteries [5–7]. To date, platinum (Pt) and Pt-based catalysts are still the most commonly used electrocatalysts for ORR, while the high cost and scarcity of Pt have been greatly hindering their scale-up application in Zn–air battery [8–10]. Hence, the development of cost-effective catalysts with satisfactory ORR activity without using Pt or other noble metals is the key subject of this field [11].

Transition metal oxides are highly promising candidates for ORR electrocatalysis due to their electronic properties in the d-state, which greatly influence the electrocatalytic activity by changing d-orbital electrons number [12]. Various oxidation states of transition metals enable the electrons transfer among the metal ions, thus contributing to their excellent ORR performances [13]. Among these transition metal oxides, manganese oxides have induced huge interests owing to its abundance, low cost, non-toxic and its various valence states (I–VI) [14, 15]. However, the large variety of Mn valence complicates the detailed electrocatalytic mechanism study and impedes further practical application. There are more than 20 polymorphs for manganese oxides [16], and their diverse chemical compositions, crystalline structures and microstructure such as morphologies and pore structures, etc., are of significance or even critical importance in affecting the catalytic activity of MnO_*x*_ [17]. Actually, the crystalline structures of manganese oxides are determined by the connection types between the [MnO_6_] units via sharing corners or edges. Bixbyite α-Mn_2_O_3_ is composed of corner-sharing [MnO_6_] octahedra [18], and a part of which will have longer apical bonds due to Jahn–Teller distortion. The layered δ-MnO_2_ has [MnO_6_]-shared edges in each layer with a variety of alkaline metal cations in between the layers, while different isomers of MnO_2_ with one-dimensional tunnel structures have varied tunnel sizes, such as 2 × 2 tunnels for α-MnO_2_, 1 × 1 tunnels for β-MnO_2_ and 1 × 2 and 1 × 1 tunnels for γ-MnO_2_ [19]. Suib and coworkers synthesized a series of manganese oxides including α-, β-, δ-MnO_2_ and amorphous MnO_2_ (AMO) via facile methods and demonstrated that the electrocatalytic activities follow the order of α-MnO_2_ > AMO > β-MnO_2_ > δ-MnO_2_ [20]. However, the electrocatalytic activities of manganese oxides are still not satisfactory because of the poor intrinsic activity and conductivity impeding the electron transfer during the ORR process, which could be enhanced by the introduction of oxygen vacancies and carbon matrix [21].

Herein, we demonstrate the fabrication of a novel Mn(II)O electrocatalyst with superior ORR performance via a facile “two-step” approach of Mn_3_O_4_ synthesis and subsequent heat treatment at varied temperatures under the reducing atmosphere. It has been found that MnO obtained by the treatment at 600 °C shows the highest ORR electrocatalytic activity of 0.895 V in half-wave potential (*E*_1/2_), which is 18 mV higher than that of commercial Pt/C (*E*_1/2_ = 0.877 V). More impressively, the ORR activity further demonstrates a significant enhancement during the long-term electrochemical test because of the in situ generation and stable existence of higher valence species in the form of Mn_5_O_8_, i.e., 2Mn_2_O_3_·MnO_2_. The production of Mn^3+^ and Mn^4+^ is responsible for the obviously enhanced O_2_ transformation ability and peroxide decomposition, respectively, further confirming that the presence of Mn^3+^/Mn^4+^ in a ratio of 2:1 in the present case is vital for the high ORR activity of MnO.

## Experimental Section

### Materials

Manganese (III) acetylacetonate (C_15_H_21_MnO_6_, 98% +) was purchased from Adamas Reagent Co., Ltd. Absolute ethyl alcohol was purchased from Shanghai Lingfeng Chemical Reagent Co., Ltd. Nafion D-520 dispersion (5 wt%) was purchased from Dupont China Holding Co., Ltd. Commercial 20 wt% Pt/C and the carbon black (XC-72) were purchased from Shanghai HEPHAS Energy Equipment Co., Ltd. All materials were used as received without further purification.

### Synthesis of Mn_3_O_4_

The Mn_3_O_4_ sample was synthesized by the hydrothermal method. First, 1 mM of manganese acetylacetonate was dissolved in 40 mL of absolute ethyl alcohol. Second, the solution was transferred into an 80 mL Teflon reaction kettle and was heated at 120 °C for 10 h. Third, the as-obtained mixture was washed with ethyl alcohol for three times and then washed with deionized water for one time by the centrifugal separation. Finally, the prepared powder was collected after the freeze drying for 12 h.

### Synthesis of MnO

The as-obtained Mn_3_O_4_ powder was thermal treated to obtain the final MnO electrocatalyst at varied temperatures (400, 500, 550, 600 and 700 °C) for 2 h in reducing gas flow (containing 5 vol% hydrogen and 95 vol% argon) in the tube furnace with the heating rate is 5 °C min^−1^. Among all samples, it has been found that 600 °C is the optimal treatment temperature for the highest catalytic activity.

### Materials Characterization

The powder X-ray diffraction (XRD) patterns were acquired on a Rigaku D/Max-2550 V X-ray diffractometer with a Cu K_α_ radiation target (40 kV, 40 mA) at a scan rate of (4°) min^−1^. X-ray photoelectron spectroscopy (XPS) signals were measured on a Thermo Fisher Scientific ESCAlab250 XPS instrument with monochromatic Al K_α_ X-rays. Binding energies of high-resolution spectra were measured after calibration, specifically, by setting C 1*s* at 284.6 eV. Spherical aberration-corrected HAADF-STEM measurements were taken on a JEM-ARM300F instrument (Shanghai Institute of Microsystem and Information Technology). Scanning electron microscope (SEM) imaging was carried out using a FEI Magellan-400 field emission scanning electron microscopy (5 kV). Transmission electron microscopy (TEM) patterns were collected using a JEM-2100F field emission transmission electron microscopy (200 kV). Electron spin resonance (ESR) signals were measured on a Bruker A300 ESR instrument.

### Electrochemical Measurements

The electrochemical measurements were carried out in 1 M KOH solution by a CH Instruments 760E electrochemical workstation using a standard three-electrode setup. During ORR test, a glassy carbon electrode (GCE) coated with catalysts, an Ag/AgCl electrode and a graphite rod were employed as the working electrode, reference electrode and counter electrode, respectively. The Ag/AgCl electrode was stored in 3 M KCl solution and rinsed with deionized water before use. All potentials were calibrated relative to the reversible hydrogen electrode (RHE) scale according to the Nernst equation (*E*_RHE_ = *E*_Ag/AgCl_ + 0.059 × pH + 0.209 V), where *E*_Ag/AgCl_ is the external potential measured against the Ag/AgCl reference electrode, which has been corrected with a reversible hydrogen electrode.

To prepare catalyst ink for ORR test, 5 mg catalyst, 5 mg carbon black and 10 μL of Nafion were dispersed in 900 μL of isopropanol and 90 μL of deionized water. Similarly, the commercial Pt/C ink was prepared by dispersing 10 mg catalyst in a solution containing 10 μL of Nafion, 900 μL of isopropanol and 90 μL of deionized water. After the mixture was sonicated for 30 min, 10 μL of homogeneous ink was pipetted onto the glassy carbon electrode. Before all electrochemical measurements, high-purity N_2_/O_2_ gas was bubbled into the solution for at least 30 min. The cyclic voltammetric (CV) measurements were performed in 1 M O_2_-saturated KOH solution at a scan rate of 100 mV s^−1^. Then, the linear sweep voltammetric (LSV) curves were measured in 1 M O_2_-saturated KOH solution at a scan rate of 10 mV s^−1^ at a rotating speed of 1600 rpm. Besides, the electrochemical impedance spectroscopy (EIS) measurements were conducted in a frequency range of 10^−2^ to 10^5^ Hz with an amplitude of 5 mV at a fixed voltage of 0.965 V (vs. RHE).

The kinetics parameters including electron transfer number (*n*) and the yield of H_2_O_2_ can be calculated from the following Eqs. (1) and (2).

1 $$ n = \frac{{4I_{\text{D}} }}{{I_{\text{D}} + I_{\text{R}} /N}} $$

2 $$ \% H_{2} O_{2} = \frac{{200I_{\text{R}} /N}}{{I_{\text{D}} + I_{\text{R}} /N}} $$

where *I*_D_ and *I*_R_ are the disk current and the ring current, respectively, and *N* is the experimental current collection efficiency by Pt ring on the ring-disk electrode, generally determined to be the value of 0.37 from the reduction of K_3_Fe(CN)_6_.

The electrochemical active surface areas of the electrode were calculated according to the Randles–Sevcik equation (Eq. (3)) [22, 23]:

3 $$ I_{\text{p}} = \, (2.69 \times 10^{5} ) \, n^{3/2} AC*D^{1/2} v^{1/2} $$

where *I*_p_ refers to the cathodic peak current, *n* is the total number of electrons transferred (*n* = 4), *A* is the effective surface area of the electrode, *D* is the diffusion coefficient for KOH = 1.9 × 10^−5^ cm S^−1^, C* is the concentration of KOH, and *v* is the scan rate.

### Zinc–Air Battery Measurements

To prepare catalyst ink for zinc–air battery test, 6 mg catalyst, 2 mg carbon black and 10 μL of Nafion were dispersed in 900 μL of isopropanol and 90 μL of deionized water. Similarly, the commercial Pt/C catalyst ink was prepared by dispersing 6 mg catalyst in a solution containing 10 μL of Nafion, 900 μL of isopropanol and 90 μL of deionized water. After the mixture was sonicated for 30 min, the as-prepared ink was pipetted onto a carbon paper substrate (the loading amount: 2 mg cm^−2^) as zinc–air battery cathode. A polished Zn foil was applied as the anode, and 6 M KOH filled with 0.2 M Zn(Ac)_2_ was applied as the electrolyte to form zincate (Zn(OH)_4_^2−^) to ensure reversible Zn electrochemical reactions at the anode. The polarization curves of zinc–air battery were recorded by a CH Instruments 760E electrochemical workstation using a standard three-electrode setup.

## Results and Discussion

### Catalyst Synthesis and Characterization

**Fig. 1:**
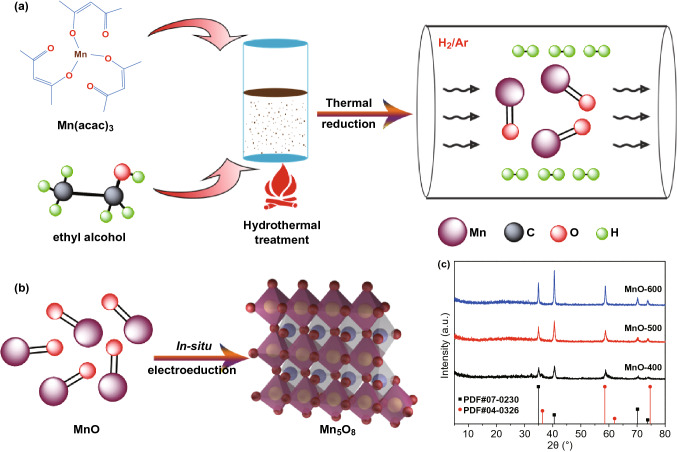
**a** Schematic illustration of the synthesis of MnO samples. **b** Structural evolution schematics of MnO_*x*_. **c** XRD patterns of MnO-T samples (*T* = 400, 500, 600 °C)

The schematic diagram of MnO synthesis is shown in Fig. 1a. First, the flower-like Mn_3_O_4_ nanosheets assemblies were readily prepared via hydrothermal treatment, which is kind of spinel oxides with mixed Mn^3+^ and Mn^2+^ valences at the tetrahedral and octahedral sites, respectively [24]. Second, the MnO electrocatalyst was obtained by the thermal-reduction of pre-synthesized Mn_3_O_4_ at varied temperatures (400, 500, 550, 600 and 700 °C) under mixed gas flow of 5 vol% hydrogen and 95 vol% argon. From the powder XRD patterns (Fig. 1c), MnO exists in the forms of both cubic (PDF#07-0230) and orthorhombic phase structures (PDF#04-0326). Furthermore, the intensities of diffraction peaks of MnO electrocatalyst clearly increase along with the increase in reduction temperature owing to the enhanced crystallinity. After measured in 1 M O_2_-saturated KOH solution for 1000 and 2000 cycles as ORR electrocatalyst, the sample MnO-600 displayed in Fig. S1 shows atypical XRD pattern of monoclinic phase Mn_5_O_8_, i.e., 2Mn_2_O_3_·MnO_2_ (PDF#39-1218), which reveals the generation and coexistence of Mn(III) and Mn(IV) in the catalyst (Fig. 1b) [25].

**Fig. 2:**
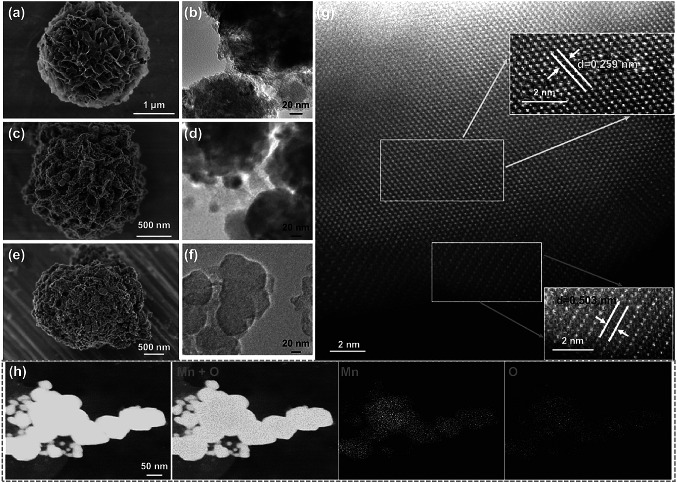
**a**, **c**, **e** SEM and **b**, **d**, **f** TEM images of Mn_3_O_4_ sample (**a**, **b**), original MnO-600 catalyst (**c**, **d**) and the correspondingly tested MnO-600 catalyst for 2000 ORR cycles (**e**, **f**). **g**, **h** Spherical aberration-corrected TEM (**g**) and TEM-EDS elemental mappings (**h**) images of MnO-600 sample

The scanning (SEM, Fig. 2a) and the transmission electron microscopic (TEM, Fig. 2b) images also demonstrate the successful synthesis of Mn_3_O_4_ nanoflowers composed of a large amount of nanosheets. After the reduction treatment at 600 °C, these nanosheets are evolved into numerous tiny nanoparticles (Fig. 2c, d). It can also be found that several big bulks appear due to the inevitable drastic grain growth during treatment at elevated temperatures. Nevertheless, as shown in Figs. S2–S4, the nanoflowers’ skeleton structures are well preserved in a wide range of temperatures (400–700 °C), which confirms the morphological stability of the MnO_*x*_ nanoflowers. In order to further prove the structure stability under electrochemical test conditions, the SEM and TEM images of MnO-600 after 1000 and 2000 ORR cycles are shown in Figs. 2e, f and S5, respectively, and it can be seen that there is no significant structure change after the test. Furthermore, the lattice fringes in spherical aberration-corrected TEM image (Fig. 2g) can be found with the measured interplanar spacings being 0.259 nm and 0.503 nm corresponding to the (111) planes of cubic MnO and (020) planes of orthorhombic MnO, respectively, consolidating the mixed cubic and orthorhombic phase structure of the sample MnO-600. In the meantime, the elemental mapping images of MnO-600 shown in Fig. 2h confirm the highly homogeneous dispersion of Mn and O elements.

**Fig. 3:**
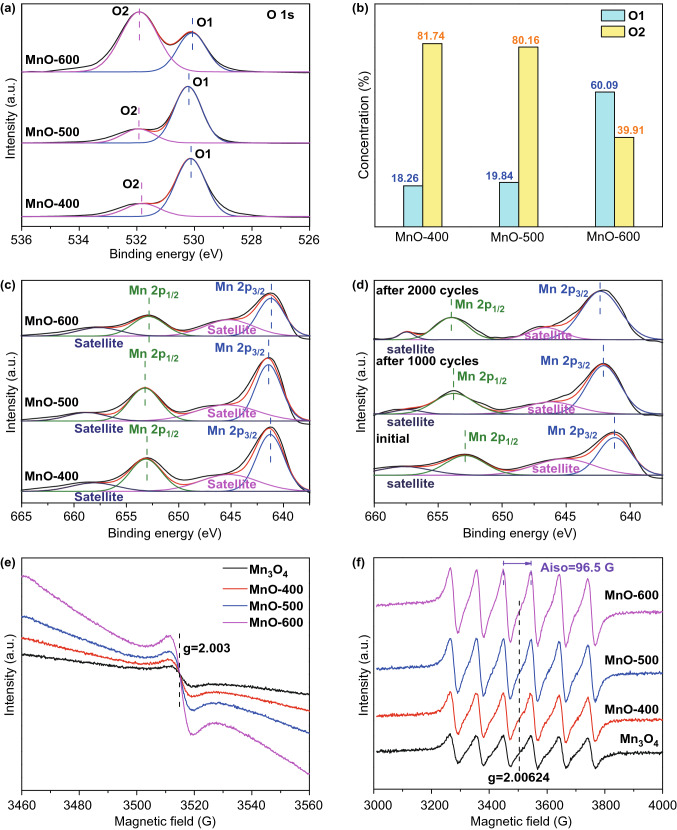
**a** XPS O 1*s* spectra among different MnO-T catalysts. **b** Contents of O1 and O2 peak for different MnO-T samples. **c**, **d** XPS Mn 2*p* spectra of the starting MnO-T catalysts (**c)** and MnO-600 of the initial and after 1000 and 2000 cycles of ORR tests (**d)**. **e**, **f** ESR measurement results of Mn_3_O_4_ and MnO-T samples for oxygen vacancies detections (**e)** and Mn valence determinations (**f)**

The large variety of Mn valence makes the structural and property investigation complicated. To clearly reveal the relationship between Mn valence and electrochemical performance, XPS measurements were performed to confirm the surface elemental composition of the catalysts. The survey spectra (Fig. S6) of Mn_3_O_4_ and MnO-T catalysts show clearly the peaks centered at around 530 and 640 eV, corresponding to O 1*s* and Mn 2*p*, respectively. Figure S7 shows the XPS Mn 2*p* spectra of Mn_3_O_4_ sample. The Mn 2*p*_1/2_ and 2*p*_3/2_ peaks of pre-synthesized Mn_3_O_4_ are at 653.05 and 641.16 eV, respectively, which is consistent with the previous reports [26]. As shown in Fig. S8, its binding energy values at 529.98 and 531.74 eV are attributed to the metal–oxygen bonding (O1) and surface oxygen defect sites (O2), respectively [27]. Besides, the comparison of XPS O 1*s* spectra among different MnO-T catalysts is shown in Fig. 3a. As the temperature gradually increases from 400 to 600 °C, the intensity ratio of O1 to O2 peak largely increases from 0.223 to 1.506, meaning an enhanced content of oxygen vacancies produced at elevated temperatures. The specific O1/O2 concentrations and the intensity ratio of O1 to O2 are displayed in Fig. 3b and Table S1.

The comparisons of XPS Mn 2*p* spectra among MnO-T (*T* = 400, 500 and 600 °C) catalysts are displayed in Fig. 3c, and the corresponding binding energy values are listed in Table S2. After the heat treatment at 400 °C, Mn 2*p*_1/2_ and 2*p*_3/2_ peaks shifted toward higher binding energy, which is due to the partial decomposition of spinel-structural Mn_3_O_4_ and the formation of Mn_2_O_3_ (III) and MnO (II) mixture. As the reduction temperature was elevated to 500 °C, two peaks further shifted toward higher binding energy owing to the higher reduction degree and more significant spinel-structure decomposition. However, when the reduction temperature was raised to 600 °C, the binding energies of two characteristic peaks began to decrease due to the disappearance of Mn^3+^, only pure MnO phase remained at this moment.

To further study how the Mn valences in manganese oxides influence the electrochemical performance of catalysts, XPS spectra of MnO-600 after 1000 and 2000 cycles of ORR tests are obtained and displayed in Fig. 3d. Compared with the pristine MnO samples, the corresponding binding energy values of Mn 2*p*_1/2_ and 2*p*_3/2_ peaks for the samples after the 1000 cycles are obviously enhanced owing to the in situ generation of high-valence Mn ions (Mn^3+^ and Mn^4+^), which is consistent with the XRD result of the formation of Mn_5_O_8_. Then, the Mn 2*p*_1/2_ and 2*p*_3/2_ peaks continue to shift toward higher binding energy levels of 653.96 and 642.3 eV (as shown in the data of Table S3), respectively, thanks to the further increase in high-valence Mn ion content after 2000 cycles, which is still in accordance with those of Mn^3+^ and Mn^4+^ mixture in the previous report [28]. In summary, the XRD patterns and XPS survey spectra all confirm the production of Mn_5_O_8_ (2Mn_2_O_3_·MnO_2_) phase during ORR tests.

Besides, the electron spin resonance (ESR) measurements were adopted to further verify the existence of oxygen vacancies and the variation of Mn valence. As shown in Fig. 3e, all samples show the resonance peaks at the g value of 2.003 attributed to oxygen vacancies [29], and the relative peak intensity increases gradually along with the increase in heat treatment temperature indicating the formation of more amount of oxygen vacancies, which is in accordance with the previous XPS results (Fig. 3a). More importantly, ESR technique is commonly used for valence analysis of transition metals and rare-earth elements owing to the incomplete occupied electron orbitals (3*d*, 4*d*, 5*d*, 4*f*…) and lone pair electrons. Generally, Mn^5+^ ions without lone pair electron display no ESR signal, whereas Mn^2+^ ions with three lone pair electrons show a typical sextet ESR pattern [30]. It can be found that all samples in Fig. 3f display such sextet patterns (*g* = 2.00624, Aiso = 96.5G) and the higher intensity sextet indicates the larger portion of Mn^2+^ in samples. As the reduction temperature reaches 600 °C, the MnO-600 sample shows the highest intensity sextet signals among ESR measurements, indicating the largest amount of Mn^2+^ in the material.

### Electrocatalytic ORR Performances

**Fig. 4:**
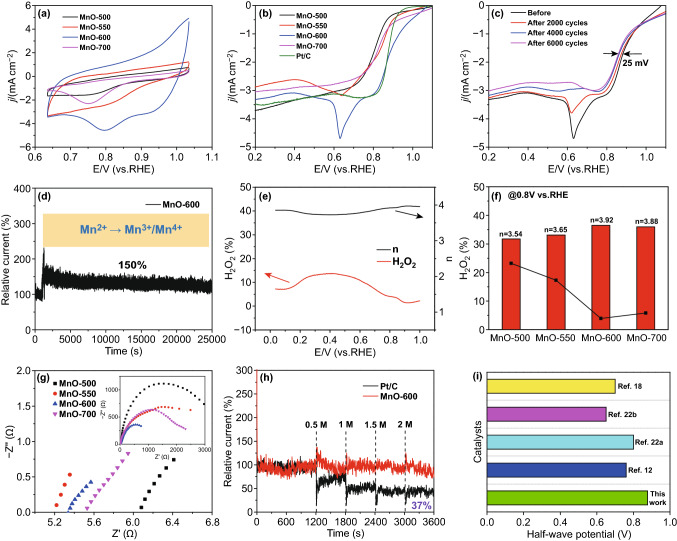
ORR performances of the prepared catalysts and their comparisons in 1 M KOH solution. **a** CV curves of MnO-T catalysts. **b** LSV curves of MnO-T catalysts at a scan rate of 10 mV/s. **c** Stability measurements for MnO-600 sample at the initial cycle and after 2000, 4000 and 6000 cycles. **d** Time-dependent current density curve at 0.7 V (vs. RHE) obtained by continuous test for 25,000 s. **e** H_2_O_2_ yields of MnO-600 at varied potentials and the corresponding electron transfer numbers. **f** H_2_O_2_ yields (dots) and the corresponding electron transfer numbers (columns) at 0.8 V (vs. RHE) for different catalysts. **g** Nyquist plots of the catalysts (inset: AC impedance curves of different catalysts at the potential of 0.965 V (vs. RHE) and in the frequency range from 10^−2^ to 10^5^ Hz). **h** Tolerance measurements to methanol at 0.7 V (vs. RHE) for Pt/C and MnO-600 catalysts. **i** Comparison of ORR performances among MnO_*x*_-based electrocatalysts in the alkaline electrolyte

The electrocatalytic performances of the synthesized catalysts for ORR were evaluated in N_2_ and O_2_-saturated 1 M KOH solutions using a rotating disk electrode (RDE) and a rotating ring-disk electrode (RRDE) system. First, the cyclic voltammetric (CV) measurements were conducted at a scan rate of 100 mV s^−1^ on MnO catalysts obtained at varied reduction temperatures to manifest the influence of sample reduction on the morphology evolution and electrochemical performance. As shown in Fig. 4a, MnO-600 exhibits a much higher electrochemical active surface area (ECSA = 0.405 cm^2^) and peak current density than the electrocatalysts treated at other temperatures. In the meantime, from the linear sweep voltammetric (LSV) polar curves (Fig. 4b), the MnO-600 catalyst clearly shows the highest ORR electrocatalytic activity of 0.895 V in its half-wave potential (*E*_1/2_), which is 18 mV higher than that of commercial Pt/C (*E*_1/2_ = 0.877 V). Furthermore, the limiting current density of MnO-600 catalyst is highly comparable to that of the state-of-the-art commercial Pt/C catalysts. The specific activity at 0.89 V versus RHE for MnO-600 is 1.52 mA cm^−2^, about 1.4 times and 4.5 times higher than that of Pt/C and MnO-500 (Fig. S9). It can also be found that there is a downward peak at around 0.623 V (vs. RHE) for MnO-600 sample, which is attributed to the valence change between Mn(II) and Mn(III) under elevated potentials. The presence of Mn(III) is beneficial to the improvement of electrocatalytic activity and thus endows the catalyst with enhanced current density. Figure S10 displays the CV curves of MnO-600 sample in N_2_ and O_2_-saturated media, which shows the presence of redox peaks and further confirms oxygen reduction activity in O_2_-saturated electrolyte.

To assess the stability of such a simple MnO-600 catalyst, a long-term cycling test between 0.6 and 1.0 V (vs. RHE) was performed. It can be found from Fig. 4c that there is no significant decrease in the ORR electrocatalytic activity even tested for 2000, 4000 and 6000 cycles, indicating the excellent ORR catalytic stability of MnO-600 catalyst during prolonged cycling tests, and the half-wave potential value has only suffered a slight loss by 25 mV. Apparently, the downward peak at around 0.623 V (vs. RHE) become gradually weakened and finally disappeared during the ORR cycling tests from the initial to 6000 cycles, indicating the completed valence change of Mn ions from 2 + to 3 +/4 + , as discussed above after the long-term cycling test. To further evaluate the catalyst stability, the chronoamperometry test was also performed. Impressively, the ORR activity will experience a significant enhancement (150% of initial current) (Fig. 4d) after the MnO electrocatalyst being measured for 1000 s under the constant applied potential (0.7 V vs. RHE), which can be explained by the production of a new component Mn_5_O_8_ as shown in the XRD patterns (Fig. S1) from MnO samples discussed above. After experiencing a period of electrochemical operation, Mn(II) ion has been fully oxidized to Mn(III) and Mn(IV), and these in situ generated Mn^3+^ and Mn^4+^ species are the key to achieve enhanced ORR activities. After that, the catalyst keeps stable at a high current density for at least 25,000 s to the end of the test, which further proves the excellent ORR stability of MnO-600 catalyst. Meanwhile, to prove the role of oxygen vacancies in MnO in elevating the ORR activity and conductivity, comparisons of LSV and ESI results between MnO-600 and MnO-purchase are shown in Figs. S12 and S13. It is clear that the MnO-600 rich in oxygen vacancies exhibits much higher ORR catalytic activity than the MnO purchased.

Moreover, the RRDE tests of the catalysts were conducted in O_2_-saturated 1 M KOH solution to calculate the H_2_O_2_ yields and the corresponding electron transfer numbers. As shown in Fig. 4e, the H_2_O_2_ yield for MnO-600 is about 10% and the corresponding electron transfer number (*n*) is close to the theoretical value of 4 at potentials ranging from 0 to 1.0 V (vs. RHE), proving a four-electron transfer pathway for ORR. The corresponding ring currents, H_2_O_2_ yields and electron transfer numbers for the other catalysts are shown in Figs. S14–S16, respectively. For further comparison, Fig. 4f displays the H_2_O_2_ yield histogram and corresponding electron transfer numbers at 0.8 V (vs. RHE) of different catalysts. Similar to the catalytic activity discussed earlier, the MnO-600 electrocatalyst shows the lowest H_2_O_2_ yield and the highest electron transfer number among all samples. The results further prove that MnO-600 electrocatalyst demonstrates better catalytic performance than the other catalysts.

The electrochemical impedance spectroscopy (EIS) measurement was conducted at 0.965 V (vs. RHE) and in the frequency range from 10^−2^ to 10^5^ Hz to elucidate the charge transfer resistance at the electrode surface. As shown in Fig. 4g and the inset, the MnO-600 electrocatalyst shows a quite low solution resistance (*R*_s_ = 5.31 Ω) and displays the smallest semicircle diameter from Nyquist plot, indicating the fastest charge transfer among all samples. Besides, the corresponding equivalence circuit for MnO-600 catalyst is shown in Fig. S17. The faster charge transfer between electrode and electrolyte results in the more significant enhancement of the catalytic activity for O_2_ reduction. On the whole, it is concluded that the MnO-600 electrocatalyst exhibits the most excellent ORR catalytic performance because of its lower charge transfer resistance and faster charge transfer speed.

Besides, the tolerance measurements to methanol crossover were conducted at 0.7 V (vs. RHE) in 1 M O_2_-saturated KOH solutions by the amperometric *i* − *t* curves (Fig. 4h). After tested for 1200 s, 0.5 M methanol into the electrolyte was added every 600 s for 4 times in all. It can be clearly seen that the current of the MnO-600 catalyst almost keeps unchanged after the additions of methanol, while the commercial Pt/C catalyst suffers substantial current density losses and remains only 37% of the initial current at the end of the tests. By comparing the ORR half-wave potentials and electron transfer numbers between the presently prepared MnO-600 catalyst and other MnO_*x*_-based alkaline electrocatalysts in the previous reports (Figs. 4i, S18 and Table S4) [31, 32], it can be found that the MnO-600 catalyst demonstrates the most excellent ORR catalytic performance.

### Zinc–Air Battery Performance

**Fig. 5:**
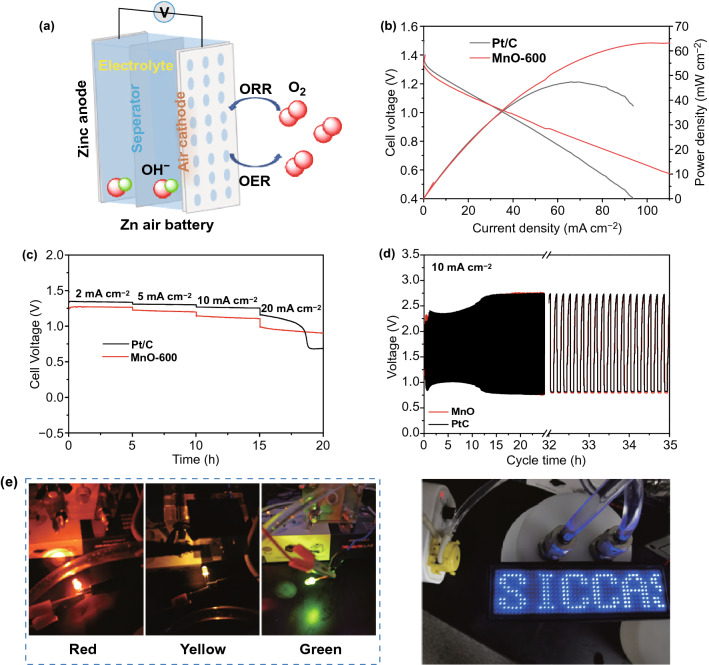
**a** Schematics of the primary configuration of a Zn–air battery. **b** Polarization curves of zinc–air battery based on MnO-600 or commercial Pt/C catalyst as cathode: cathode area: 3 cm^2^; loading amount: 2 mg cm^−2^; temperature: ~ 20 °C; **c** longtime durability of zinc–air battery by using MnO-600 or commercial Pt/C as cathode catalyst at the current densities of 2, 5, 10 and 20 mA cm^−2^. **d** Galvanostatic discharge–charge cycling curve at 10 mA cm^−2^ of the zinc–air battery using MnO-600 or Pt/C catalyst. **e** Photographs of different light-emitting diode (1.5 V, 2–2.2 V)

To further demonstrate the extra-high ORR catalytic activity of the as-synthesized MnO (II) electrocatalyst, zinc–air batteries were assembled as exemplified in Fig. 5a, in which a carbon paper pre-coated with the ORR catalyst MnO-600, or commercial Pt/C catalyst, was used as an air cathode, in coupling with a Zn anode and a glassy fiber membrane soaked with aqueous 6 M KOH electrolyte as the separator. Figure 5b shows the polarization and power density curves of air-breathing zinc–air battery with a working electrode area of 3 cm^2^ and catalyst loading amount of 2 mg cm^−2^, which were operated at room temperature of ~ 20 °C. It can be seen that the peak current density and peak power density of MnO-600 are as high as 102.7 mA cm^−2^ and 63.2 mW cm^−2^, respectively, which are markedly higher than those of commercial Pt/C (peak current density = 69.2 mA cm^−2^; peak power density = 47.4 mW cm^−2^) under identical test conditions.

It can be confirmed from Fig. 5c that the cell voltage of MnO-600 catalyst keeps as stable as commercial Pt/C at the relatively low applied currents in the range of 2–10 mA cm^−2^. When the applied current density reaches 20 mA cm^−2^, the commercial Pt/C experiences a rapid deactivation, while MnO-600 still retains a cell voltage platform, suggesting the much better stability of MnO-600 catalyst under high current density. Moreover, durability of the batteries was further evaluated using galvanostatic recurrent pulse method at a constant current density of 10 mA cm^−2^ and a discharge/recharge cyclic duration of 10 min (5 min each) (Fig. 5d). It is obvious that the Zn–air battery using MnO-600 as cathode catalyst is highly rechargeable and shows negligible voltage decay during 35 h of the cyclic test, which is highly comparable to that of commercial Pt/C benchmark. As an example for practical application (Fig. 5e), multiple Zn–air batteries were connected in series to power different light-emitting diode (2–2.2 V) and the LED display (1.5 V). A real-time LED video powered by Zn–air batteries by using MnO-600 as cathode catalyst is shown in Supplement Movie. All these operations clearly demonstrate that MnO-600 catalyst is a promising catalyst for advanced energy storage and conversion technologies.

## ORR Mechanism Probing

Generally, the electrocatalytic oxygen reduction in alkaline media could proceed via either a direct four-electron pathway producing hydroxide (OH^−^) or a two-step (two-electron + two-electron) pathway producing hydroperoxide (HO_2_^−^) groups. The detailed reaction equations are displayed in Eqs. (4)–(7) [33, 34].

4 $$ {\text{O}}_{2} + 2{\text{H}}_{2} {\text{O}} + 4{\text{e}}^{ - } = 4{\text{OH}}^{ - } \quad \left( {\text{four - electron pathway}} \right) $$

or

5 $$ {\text{O}}_{2} + {\text{H}}_{2} {\text{O}} + 2{\text{e}}^{ - } = {\text{HO}}_{2}^{ - } + {\text{OH}}^{ - } \quad \left( {\text{two - electron pathway}} \right) $$

6 $$ {\text{HO}}_{2}^{ - } + {\text{H}}_{2} {\text{O}} + 2{\text{e}}^{ - } = 3{\text{OH}}^{ - } \quad \left( {\text{two - electron pathway}} \right) $$

7 $$ \left( {{\text{or}}\,{\text{HO}}_{2}^{ - } = {\text{OH}}^{ - } + 1/2{\text{O}}_{2} } \right) $$

It has been believed that pure Mn^2+^ or Mn^4+^ species are not ORR kinetic-favorable owing to the occurrence of a portion of two-electron reduction pathway. Nevertheless, the catalyst will experience an oxygen reduction pathway via a quasi-four-electron pathway after the introduction of a small amount of Mn^3+^ [35]. However, Mn^3+^ would be unlikely to exist stably due to the inevitable Jahn–Teller distortion [36]. According to the Jahn-Tell effect, unstable Mn^3+^ will be disproportionated to Mn^2+^ and Mn^4+^ (2 Mn^3+^ = Mn^2+^ + Mn^4+^) at pH < 9 [37]. In this study, Mn^3+^ was in situ generated which could exist stably in 1 M KOH solutions (pH = 14) via the electrochemical method under the constant applied potential.

**Fig. 6:**
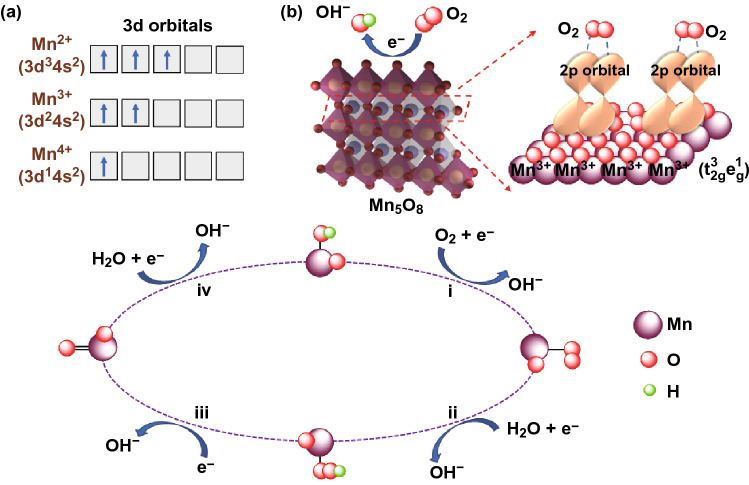
**a** Occupied states of 3*d* orbitals for Mn^2+^, Mn^3+^ and Mn^4+^. **b** Lattice structure of Mn_5_O_8_ (yellow: Mn^4+^; blue: Mn^3+^) and the ball-stick model of the O_2_ molecule adsorptions on the surface. **c** Possible four-electron reaction mechanism for MnO_*x*_ in ORR process

Besides the effect on electron transfer number, the non-negligible role of Mn^3+^ in ORR catalysis can also be found on facilitating the interaction between the electronic structure and absorbed oxygen. The coexistence of Mn^3+^ and Mn^4+^ has been reported to promote the cleavage of O–O bonds and thus ensures the rapid reduction of O_2_ to OH^−^. The trivalent Mn ion adopts a high-spin d^4^ configuration (t_2g_^3^e_g_^1^) [38], whereas the produced Mn^2+^ and Mn^4+^ (due to the charge disproportionation of Mn^3+^) possess non-degenerated t_2g_^3^e_g_^2^ and t_2g_^3^e_g_^0^ configurations, respectively (Fig. 6a) [37]. In a truncated octahedral environment, the antibonding orbitals of Mn^3+^ will overlap directly with that of top-absorbed O species thus to influence the bonding strength of O_2_ onto Mn^3+^ via the filling status (Fig. 6b) [19]. According to the wildly accepted four-step proton-coupled electron transfer reaction mechanism (Fig. 6c), the four-electron process for the ORR catalyzed by MnO_*x*_ can be schemed as [39]: (1) O_2_ is absorbed and transformed into OO_2_^−^ on a Mn site by displacing the OH^−^ group originally adhered on the catalyst surface in the alkaline solution; (2) the OO_2_^−^ group is protonated to form OOH^−^; (3) an OH^−^ group is removed from OOH^−^, leaving a superoxo O_2_^−^ group on the previous Mn site; (4) the O_2_^−^ group is again protonated to form OH^−^ thus to rebuild the initial hydroxyl-covered surface of MnO_*x*_. This is a complete cycle of ORR accompanying the reversible component change on the MnO_*x*_ catalyst surface during the oxygen reduction. The corresponding reactions are formulated as follows:

8 $$ {\text{O}}_{2} + {\text{Mn}} - {\text{OH}} + {\text{e}}^{ - } = {\text{Mn}} - {\text{OO}}^{*} + {\text{OH}}^{ - } $$

9 $$ {\text{Mn}} - {\text{OO}}^{*} + {\text{H}}_{2} {\text{O}} + {\text{e}}^{ - } = {\text{Mn}} - {\text{OOH}} + {\text{OH}}^{ - } $$

10 $$ {\text{Mn}} - {\text{OOH}} + {\text{e}}^{ - } = {\text{Mn}} - {\text{O}} + {\text{OH}}^{ - } $$

11 $$ {\text{Mn}} - {\text{O}} + {\text{H}}_{2} {\text{O}} + {\text{e}}^{ - } = {\text{Mn}} - {\text{OH}} + {\text{OH}}^{ - } $$

From the four ORR steps taking place on the MnO, i.e., the oxygen adsorption to replace OH^−^ group at the Mn site (step 1) is regarded as the rate-determining step in the ORR kinetics on metal oxides. On the one hand, the synthesized MnO-600 catalyst possesses the largest amount of oxygen vacancies in the prepared materials according to the XPS O1*s* spectra and ESR result, and thus, the presence of oxygen vacancies will accelerate the adsorption of O_2_ molecules to promote step (1), resultantly facilitating the ORR activity elevation. On the other hand, the in situ generated large quantity of Mn^3+^ ions during the electrochemical test of MnO will lead to much weakened oxygen adsorption because of the existence of electron in the orbitals of Mn^3+^, thus favoring the cleavage/desorption of OH^−^ groups from the Mn sites and accelerating the steps (1) to (4), consequently resulting in largely enhanced ORR kinetics.

In addition, Mn^4+^ is proved to be highly active for peroxide decomposition [40]. The in situ generated Mn^4+^ species in Mn_5_O_8_ of MnO-600 catalyst will facilitate the reaction (10) and accelerate the ORR process during the electrochemical tests. Thus, the excellent ORR performance of synthesized MnO-600 can be attributed to the synergistic catalytic effect among the presence of oxygen vacancies and the in situ generated Mn^3+^ and Mn^4+^ species, in which the oxygen vacancies accelerate the adsorption of O_2_ molecules, Mn^3+^ catalyze ORR and Mn^4+^ catalyze peroxide decomposition in a synergetic way, i.e., the oxygen vacancies, Mn^3+^ and Mn^4+^ ions function successively in the ORR catalysis [41], resulting in overall enhanced ORR catalytic activity.

## Conclusion

A simple noble metal-free Mn(II)O electrocatalyst with excellent ORR performance has been fabricated via a facile “two-step” strategy. The excellent ORR electrocatalytic performance has been obtained on the MnO catalyst synthesized by heat-reducing the starting Mn_3_O_4_ at 600 °C, which exhibits a half-wave potential (*E*_1/2_) of as high as 0.895 V, 18 mV higher than that of commercial Pt/C (*E*_1/2_ = 0.877 V). Besides, the marked Zn–air battery performances in terms of peak current density, peak power density and durability have been achieved by using the MnO-600 as the cathode electrocatalyst. More impressively, the ORR activity can be significantly enhanced during the long-term electrochemical test owing to the in situ generation and stable existence of higher valence manganese ions in the form of 2Mn_2_O_3_·MnO_2_. According to the mechanism analysis for the ORR reaction pathway, a synergetic catalytic effect has been proposed, in which the oxygen vacancies, and in situ generated Mn^3+^ and Mn^4+^ species function successively in the ORR catalysis, resulting in the much enhanced catalytic activity. This work not only broadens our horizon for constructing high-performance noble metal-free electrocatalyst by employing multivalence transition metal oxides, but also provides an in-depth mechanistic probing on the relationship between Mn valence and electrochemical performance.

## Electronic supplementary material

Below is the link to the electronic supplementary material.

Supplementary material 1 (PDF 1357 kb)

Supplementary material 2 (MP4 1087 kb)

## References

[1] Chong L, Wen J, Kubal J, Sen F, Zou J (2018). Ultralow-loading platinum-cobalt fuel cell catalysts derived from imidazolate frameworks. Science.

[2] Wan X, Liu X, Li Y, Yu R, Zheng L (2019). Fe–N–C electrocatalyst with dense active sites and efficient mass transport for high-performance proton exchange membrane fuel cells. Nat. Catal..

[3] Zhang J, Zhao Z, Xia Z, Dai L (2015). A metal-free bifunctional electrocatalyst for oxygen reduction and oxygen evolution reactions. Nat. Nanotech..

[4] Kong F, Fan X, Kong A, Zhou Z, Zhang X, Shan Y (2018). Covalent phenanthroline framework derived FeS@Fe_3_C composite nanoparticles embedding in N-S-codoped carbons as highly efficient trifunctional electrocatalysts. Adv. Funct. Mater..

[5] Xiao M, Zhu J, Feng L, Liu C, Xing W (2015). Meso/macroporous nitrogen-doped carbon architectures with iron carbide encapsulated in graphitic layers as an efficient and robust catalyst for the oxygen reduction reaction in both acidic and alkaline solutions. Adv. Mater..

[6] Yu P, Wang L, Sun F, Xie Y, Liu X (2019). Co Nanoislands rooted on Co–N–C nanosheets as efficient oxygen electrocatalyst for Zn–air batteries. Adv. Mater..

[7] Liu X, Wang L, Yu P, Tian C, Sun F (2018). A stable bifunctional catalyst for rechargeable zinc–air batteries: iron–cobalt nanoparticles embedded in a nitrogen-doped 3D carbon matrix. Angew. Chem. Int. Ed..

[8] Tian X, Zhao X, Su Y, Wang L, Wang H (2019). Engineering bunched Pt–Ni alloy nanocages for efficient oxygen reduction in practical fuel cells. Science.

[9] Qin Q, Jang H, Li P, Yuan B, Liu X, Cho J (2019). A tannic acid-derived N-, P-codoped carbon-supported iron-based nanocomposite as an advanced trifunctional electrocatalyst for the overall water splitting cells and zinc–air batteries. Adv. Energy Mater..

[10] Li P, Jang H, Zhang J, Tian M, Chen S (2019). A metal-free N and P-codoped carbon nanosphere as bifunctional electrocatalyst for rechargeable zinc–air batteries. ChemElectroChem.

[11] Nie Y, Li L, Wei Z (2015). Recent advancements in Pt and Pt-free catalysts for oxygen reduction reaction. Chem. Soc. Rev..

[12] Bhargava A, Chen C, Dhaka K, Yao Y, Nelson A (2019). Mn cations control electronic transport in spinel Co_*x*_Mn_3–*x*_O_4_ nanoparticles. Chem. Mater..

[13] Ge X, Sumboja A, Wuu D, An T, Li B (2015). Oxygen reduction in alkaline media: from mechanisms to recent advances of catalysts. ACS Catal..

[14] Lee S, Nam G, Sun J, Lee JS, Lee HW (2016). Enhanced intrinsic catalytic activity of λ-MnO_2_ by electrochemical tuning and oxygen vacancy generation. Angew. Chem. Int. Ed..

[15] Huang Y, Mou J, Liu W, Wang X, Dong L, Kang F, Xu C (2019). Novel insights into energy storage mechanism of aqueous rechargeable Zn/MnO_2_ batteries with participation of Mn^2+^. Nano-Micro Lett..

[16] Robinson DM, Go YB, Mui M, Gardner G, Zhang Z (2013). Photochemical water oxidation by crystalline polymorphs of manganese oxides: structural requirements for catalysis. J. Am. Chem. Soc..

[17] Asif M, Aziz A, Dao A, Hakeem A, Wang H (2015). Real-time tracking of hydrogen peroxide secreted by live cells using MnO_2_ nanoparticles intercalated layered doubled hydroxide nanohybrids. Anal. Chim. Acta.

[18] Geller S (1971). Structures of α-Mn_2_O_3_, (Mn_0.983_Fe_0.017_)_2_O_3_ and (Mn_0.37_Fe_0.63_)_2_O_3_ and relation to magnetic ordering. Acta Crystallogr. B.

[19] Stoerzinger KA, Risch M, Han B, Shao-Horn Y (2015). Recent insights into manganese oxides in catalyzing oxygen reduction kinetics. ACS Catal..

[20] Meng Y, Song W, Huang H, Ren Z, Chen SY, Suib SL (2014). Structure-property relationship of bifunctional MnO_2_ nanostructures: highly efficient, ultra-stable electrochemical water oxidation and oxygen reduction reaction catalysts identified in alkaline media. J. Am. Chem. Soc..

[21] Tian H, Cui X, Zeng L, Su L, Song Y, Shi J (2019). Oxygen vacancy-assisted hydrogen evolution reaction of the Pt/WO_3_ electrocatalyst. J. Mater. Chem. A.

[22] Aziz A, Asif M, Azeem M, Ashraf G, Wang Z, Xiao F, Liu H (2019). Self-stacking of exfoliated charged nanosheets of LDHs and graphene as biosensor with real-time tracking of dopamine from live cells. Anal. Chim. Acta.

[23] Asif M, Aziz A, Ashraf G, Wang Z, Wang J (2018). Facet-inspired core–shell gold nanoislands on metal oxide octadecahedral heterostructures: high sensing performance toward sulfide in biotic fluids. ACS Appl. Mater. Interfaces.

[24] Liu Y, Ying Y, Fei L, Liu Y, Hu Q (2019). Valence engineering via selective atomic substitution on tetrahedral sites in spinel oxide for highly enhanced oxygen evolution catalysis. J. Am. Chem. Soc..

[25] Oswald HR (1965). Crystal data of Mn_5_O_8_ and Cd_2_Mn_3_O_8_. Nature.

[26] Gorlin Y, Chung CJ, Nordlund D, Clemens BM, Jaramillo TF (2012). Mn_3_O_4_ supported on glassy carbon: an active non-precious metal catalyst for the oxygen reduction reaction. ACS Catal..

[27] Choi Y, Lim D, Oh E, Lim C, Baeck SH (2019). Effect of proton irradiation on electrocatalytic properties of MnO_2_ for oxygen reduction reaction. J. Mater. Chem. A.

[28] Shan X, Charles DS, Lei Y, Qiao R, Wang G (2016). Bivalence Mn_5_O_8_ with hydroxylated interphase for high-voltage aqueous sodium-ion storage. Nat. Commun..

[29] Wan J, Chen W, Jia C, Zheng L, Dong J (2018). Defect effects on TiO_2_ nanosheets: stabilizing single atomic site Au and promoting catalytic properties. Adv. Mater..

[30] Er G, Ishida S, Takeuchi N (1999). Investigations of the electrical property, diffuse reflectance and ESR spectra of the La-(Fe, Mn)-codoped PTCR BaTiO_3_ annealed in reducing atmosphere. J. Mater. Sci..

[31] Kang B, Jin X, Oh SM, Patil SSB, Kim MG, Kim SH, Hwang SJ (2018). An effective way to improve bifunctional electrocatalyst activity of manganese oxide via control of bond competition. Appl. Catal. B.

[32] Cui X, Hua Z, Chen L, Zhang X, Chen H, Shi J (2016). Manganese oxide nanorod-decorated mesoporous ZSM-5 composite as a precious-metal-free electrode catalyst for oxygen reduction. Chemsuschem.

[33] Hong WT, Risch M, Stoerzinger KA, Grimaud A, Suntivich J, Shao-Horn Y (2015). Toward the rational design of non-precious transition metal oxides for oxygen electrocatalysis. Energy Environ. Sci..

[34] Wang DW, Su D (2014). Heterogeneous nanocarbon materials for oxygen reduction reaction. Energy Environ. Sci..

[35] Zhang B, Chen H, Daniel Q, Philippe B, Yu F (2017). Defective and “c-disordered” hortensia-like layered MnO_*x*_ as an efficient electrocatalyst for water oxidation at neutral pH. ACS Catal..

[36] Zhang Q, Didier C, Pang WK, Liu Y, Wang Z (2019). Structural insight into layer gliding and lattice distortion in layered manganese oxide electrodes for potassium-ion batteries. Adv. Energy Mater..

[37] Takashima T, Hashimoto K, Nakamura R (2012). Inhibition of charge disproportionation of MnO_2_ electrocatalysts for efficient water oxidation under neutral conditions. J. Am. Chem. Soc..

[38] Sakai N, Sasaki T (2005). Photocurrent generation from semiconducting manganese oxide nanosheets in response to visible light. J. Phys. Chem. B.

[39] Suntivich J, Gasteiger HA, Yabuuchi N, Nakanishi H, Goodenough JB, Shao-Horn Y (2011). Design principles for oxygen-reduction activity on perovskite oxide catalysts for fuel cells and metal-air batteries. Nat. Chem..

[40] Chinnadurai D, Nallal M, Kim H, Li O, Park K, Prabakar K (2020). Mn^3+^ active surface site enriched manganese phosphate nano-polyhedrons for enhanced bifunctional oxygen electrocatalyst. Chemcatchem.

[41] Shi J (2013). On the synergetic catalytic effect in heterogeneous nanocomposite catalysts. Chem. Rev..

