# Supplementary material for: In Situ Electrochemical Mn(III)/Mn(IV) Generation of Mn(II)O Electrocatalysts for High-Performance Oxygen Reduction
Source: Nanomicro Lett. 2020 Aug 11;12:161. doi: 10.1007/s40820-020-00500-7 (PMC7770805; doi:10.1007/s40820-020-00500-7)
Supplement: Supplementary file 1 — Supplementary material 1 (PDF 1357 kb) [file 40820_2020_500_MOESM1_ESM.pdf]

Supporting Information for

In-Situ Electrochemical Mn(III)/Mn(IV) Generation of Mn(II)O Electrocatalysts for High-Performance Oxygen Reduction

Han Tian[1, 2], Liming Zeng[4], Yifan Huang[5], Zhonghua Ma[6], Ge Meng[1, 2], Lingxin Peng[1, 2], Chang Chen[1, 2], Xiangzhi Cui[1, 2, 3, *], Jianlin Shi[1, 2, *]

[1]State Key Lab of High Performance Ceramics and Superfine Microstructure, Shanghai Institute of Ceramics, Chinese Academy of Sciences, Shanghai, 200050, People's Republic of China

[2]Center of Materials Science and Optoelectronics Engineering, University of Chinese Academy of Sciences, Beijing 100049, People's Republic of China

[3]School of Chemistry and Materials Science, Hangzhou Institute for Advanced Study, University of Chinese Academy of Sciences, Hangzhou 310021, People's Republic of China

[4]College of Chemistry and Molecular Sciences, Hubei Key Lab of Electrochemical Power Sources, Wuhan University, 430072, People's Republic of China

[5]Wuhan University of Science and Technology, Wuhan 430081, People's Republic of China

[6]College of Material Science and Engineering, Donghua University, Shanghai 201620, People's Republic of China

*Corresponding authors. E-mail: jlshi@mail.sic.ac.cn (Jianlin Shi); cuixz@mail.sic.ac.cn (Xiangzhi Cui)

Supplementary Figures and Tables

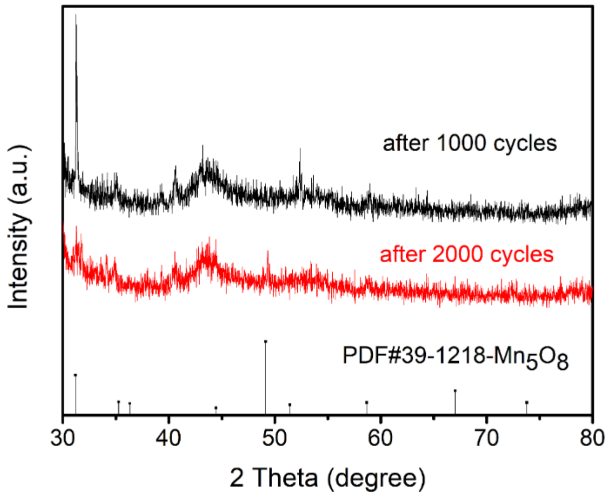

Fig. S1 XRD patterns of MnO-600 sample after 1000 and 2000 cycles of ORR test

To obtain the sufficient sample for XRD measurement after ORR test, we use carbon fiber cloth as a substrate carrying MnO-600 sample, which was directly used as working electrode. Thus, the above XRD patterns contain some signals of carbon fiber cloth inevitably.

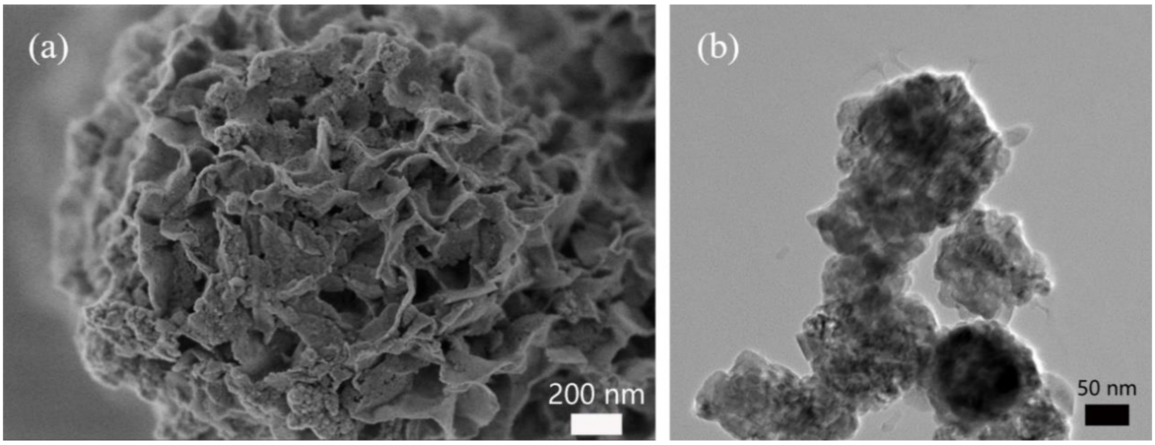

Fig. S2 (**a**) SEM and (**b**) TEM images of MnO-400 catalyst

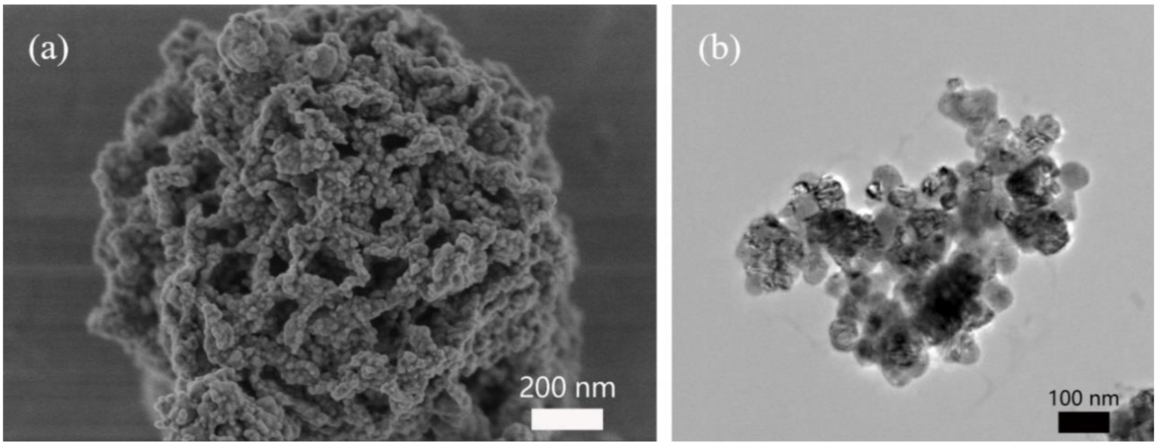

Fig. S3 (**a**) SEM and (**b**) TEM images of MnO-500 catalyst

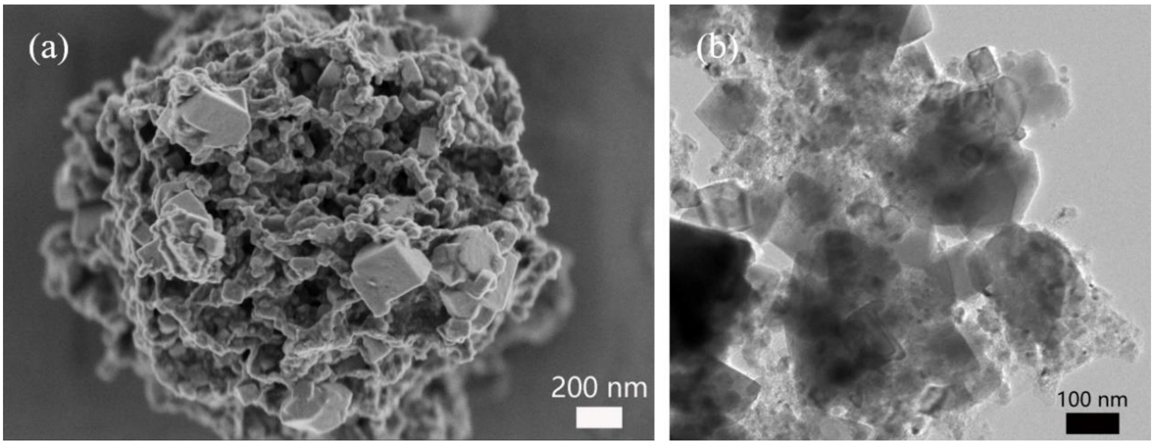

Fig. S4 (**a**) SEM and (**b**) TEM images of MnO-700 catalyst

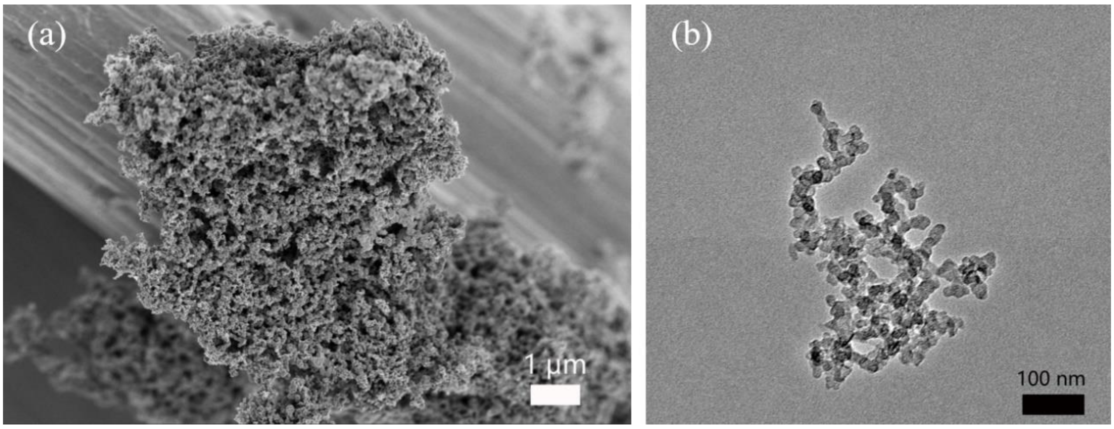

Fig. S5 (**a**) SEM and (**b**) TEM images of MnO-600 sample after 1000 cycles of ORR test

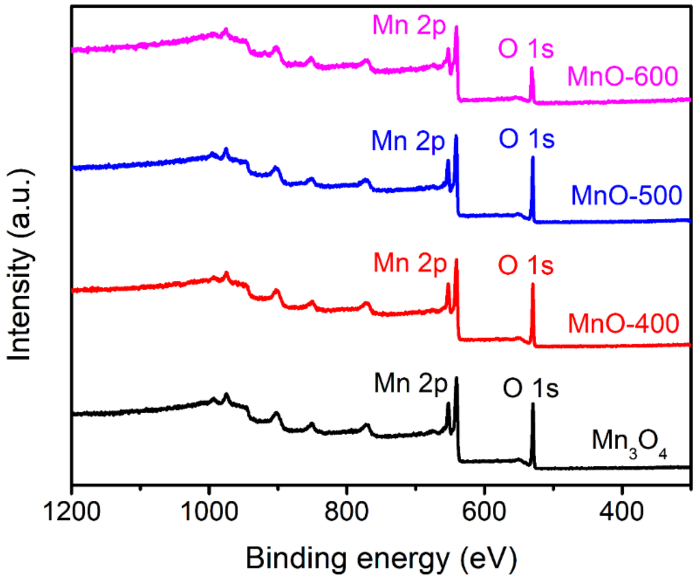

Fig. S6 XPS survey spectra of pre-synthesized Mn_3O_4 and MnO-T catalysts

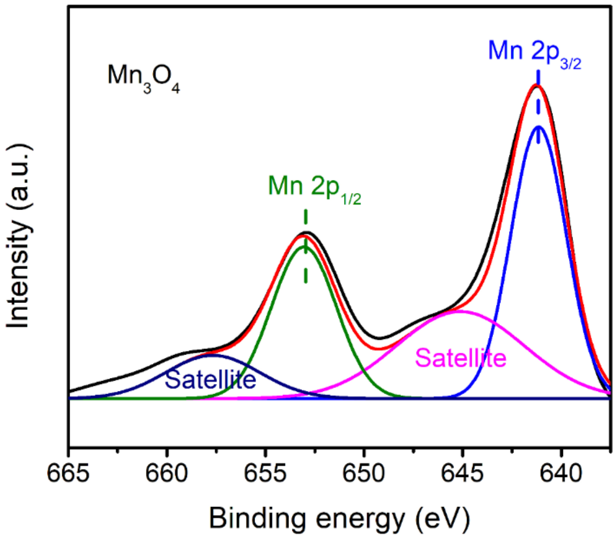

Fig. S7 XPS Mn 2p spectra of Mn_3O_4 intermediate product

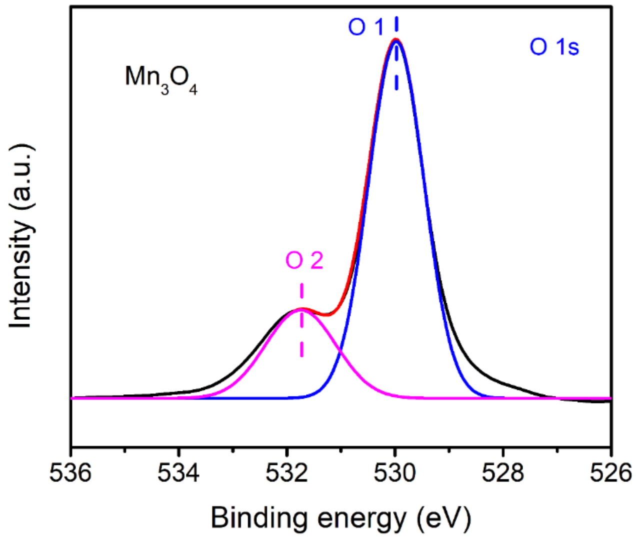

Fig. S8 Detailed XPS O 1s spectra of pre-synthesized Mn_3O_4 sample

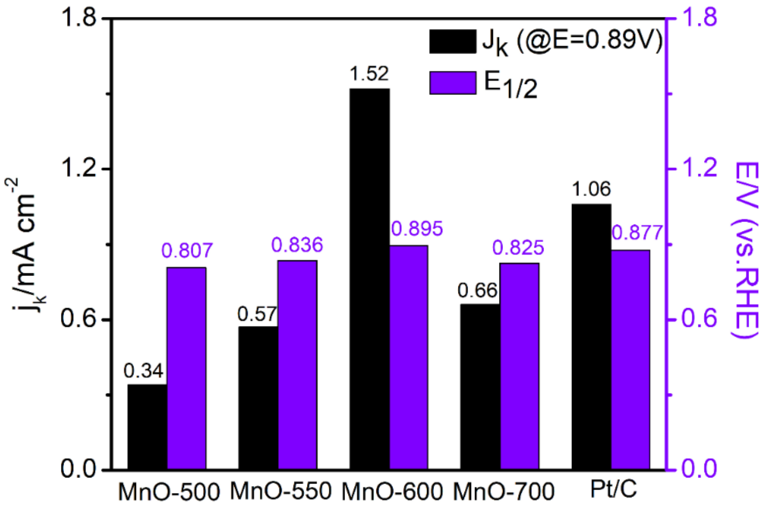

Fig. S9 Specific activity at 0.89 V and $E_{1/2}$

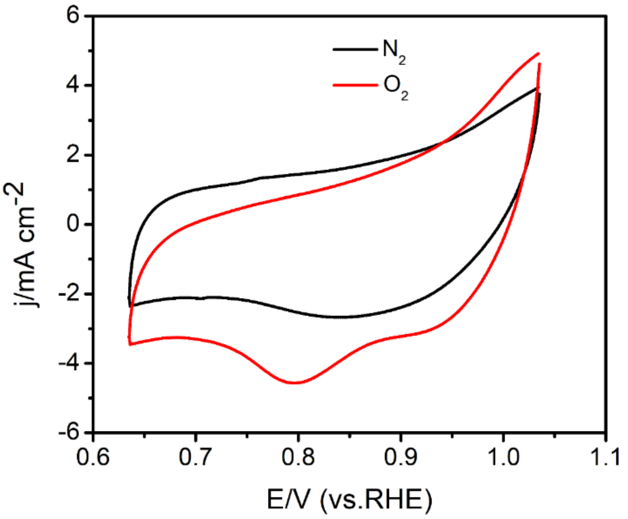

Fig. S10 CV curves of MnO-600 catalyst under N_2 and O_2-saturated conditions

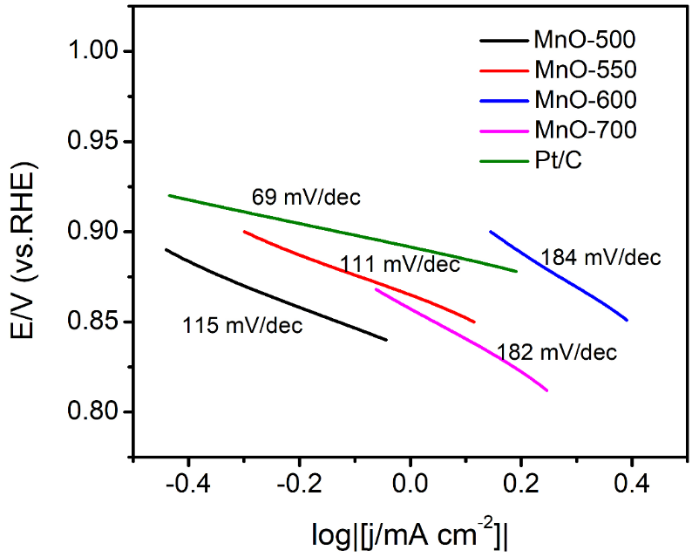

Fig. S11 Linear portions of the Tafel plots for all samples

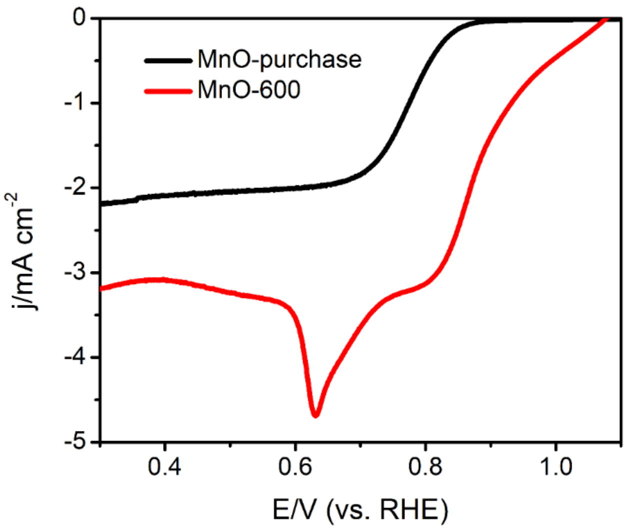

Fig. S12 Comparison of LSV curves for MnO-600 and MnO-purchase

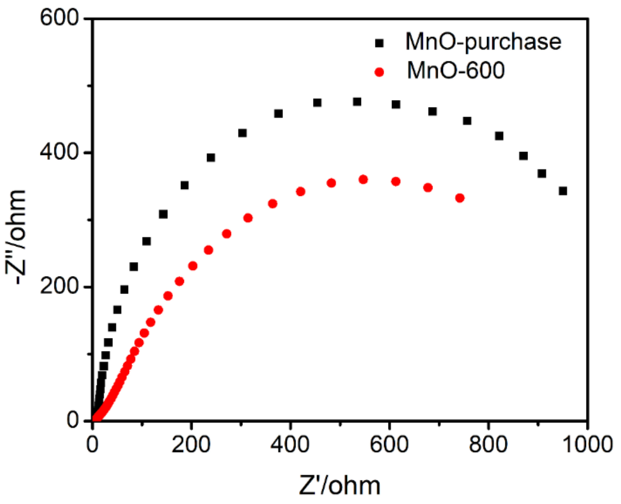

Fig. S13 Comparison of ESI curves for MnO-600 and MnO-purchase

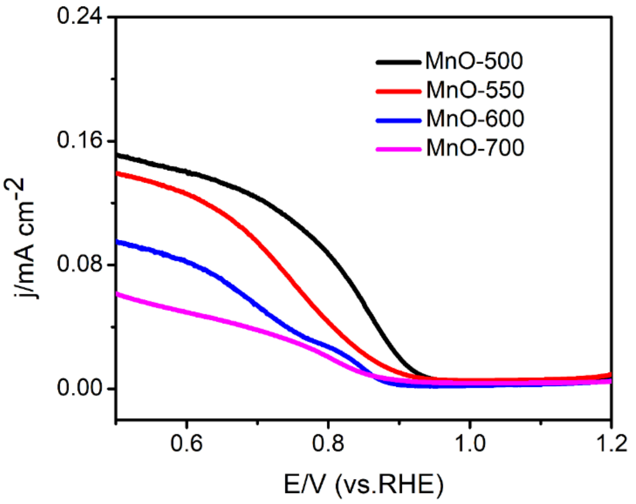

Fig. S14 LSV curves of ring currents for MnO-T samples in ORR process in KOH

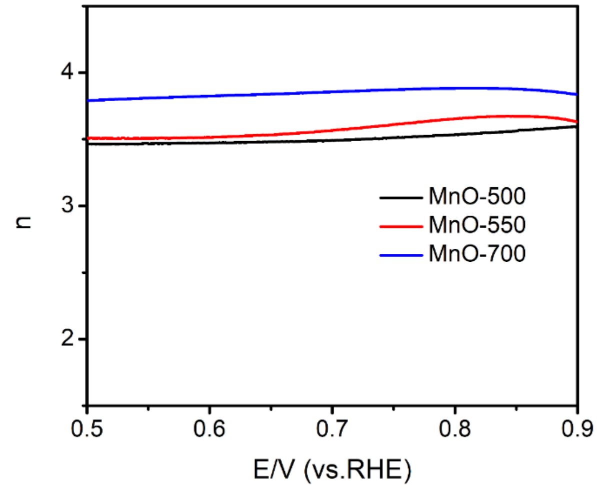

Fig. S15 Electron transfer numbers for MnO-500, MnO-550 and MnO-700 samples in ORR process in KOH

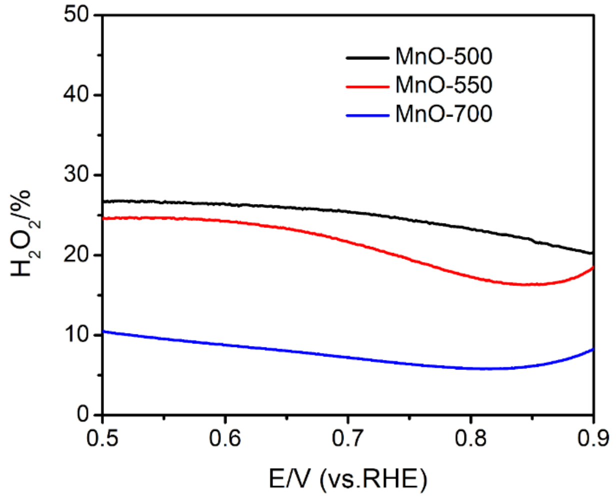

Fig. S16 H_2O_2 yields for MnO-500, MnO-550 and MnO-700 samples in ORR process in KOH

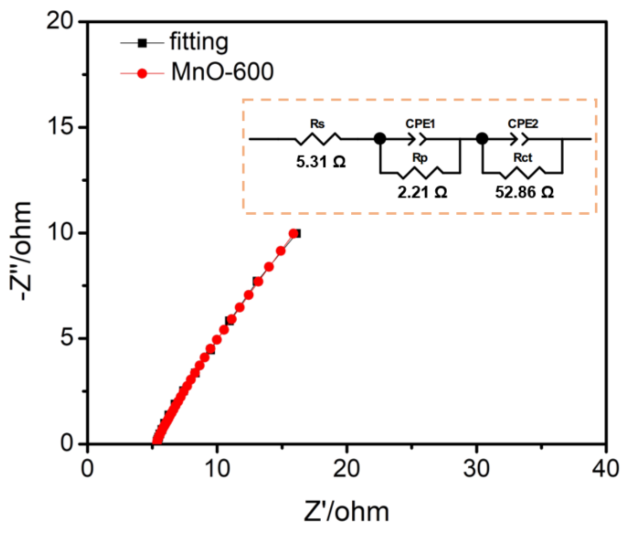

Fig. S17 Corresponding equivalence circuit for MnO-600 catalyst

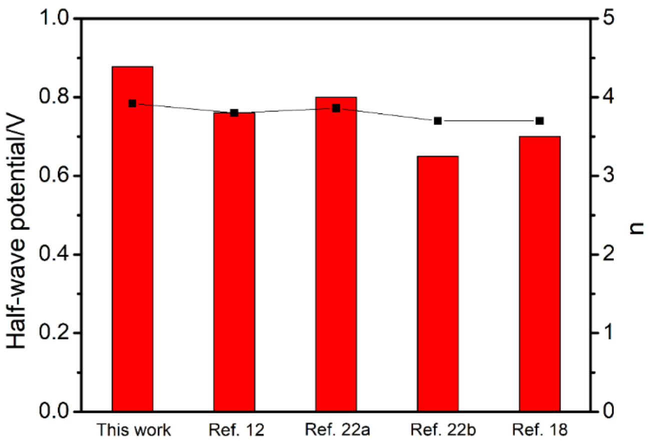

Fig. S18 Comparison of ORR performances among MnO_x-based electrocatalysts in the alkaline electrolyte

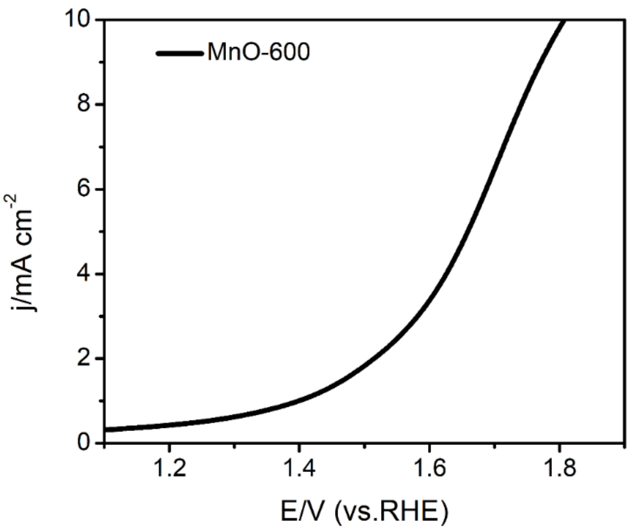

Fig. S19 LSV curve of MnO-600 for OER in 1 M KOH

Table S1 The ratio of O1 to O2 peak in XPS O 1s spectra for MnO-T samples (corresponding to **Fig. 3a**)

	%Area of O1	%Area of O2	Ratio of O1 to O2
MnO-400	18.26	81.74	0.223
MnO-500	19.84	70.16	0.248
MnO-600	60.09	39.91	1.506

Table S2 Energy levels of Mn and O species in XPS survey spectra of all samples

Samples/eV	Mn $2p_{3/2}$	Mn $2p_{3/2}$ satellite	Mn $2p_{1/2}$	Mn $2p_{1/2}$ satellite	O 1	O2
Mn_3O_4	657.67	653.05	645.12	641.16	529.98	531.74
MnO-400	658.07	653.14	645.21	641.27	530.13	531.81
MnO-500	658.79	653.25	645.41	641.44	530.22	531.94
MnO-600	657.62	652.88	645.18	641.21	530.09	531.91

Table S3 Energy levels of manganese species in XPS survey spectra for MnO-600 catalyst after 1000 and 2000 ORR cycles

Samples/eV	Mn $2p_{3/2}$	Mn $2p_{3/2}$ satellite	Mn $2p_{1/2}$	Mn $2p_{1/2}$ satellite
MnO-600-after 1000	657.36	653.77	646.18	642.07
MnO-600-after 2000	657.48	653.96	646.58	642.30

Table S4 Performances of MnO_x-based electrocatalysts for ORR in alkaline electrolyte. We here use the data of relatively high catalytic activities of the catalysts reported with in respective references

Materials	Activity (half-wave potential)	Reference No.
MnO	0.877 V	This work
α-MnO_2	0.76 V	J. Am. Chem. Soc., 2014, 136, 11452−11464 DOI: 10.1021/ja505186m
$Mn_{0.85}Ru_{0.15}O$ nanowires	0.8 V	Appl. Catal. B, 2018, 236, 107–116 DOI: 10.1016/j.apcatb.2018.05.010
MnO_2/m-ZSM-5	0.65 V	ChemSusChem, 2016, 9, 1010 – 1019 DOI: 10.1002/cssc.201600012
MnO_2-14MeV-sol	0.7 V	J. Mater. Chem. A, 2019, 7, 11659-11664 DOI: 10.1039/C9TA03879E
